# Qualitative analysis of small (≤2 cm) regenerative nodules, dysplastic nodules and well-differentiated HCCs with gadoxetic acid MRI

**DOI:** 10.1186/s12880-016-0165-5

**Published:** 2016-11-11

**Authors:** Michele Di Martino, Michele Anzidei, Fulvio Zaccagna, Luca Saba, Sandro Bosco, Massimo Rossi, Stefano Ginanni Corradini, Carlo Catalano

**Affiliations:** Sapienza, University of Rome, Rome, Italy

**Keywords:** Regenerative nodule, Dysplastic nodule, Well-differentiated HCC, Magnetic resonance, Liver specific contrast agent

## Abstract

**Background:**

The characterization of small lesions in cirrhotic patients is extremely difficult due to the overlap of imaging features among different entities in the step-way of the hepatocarcinogenesis. The aim of our study was to evaluate the role of gadoxetic-acid MRI in the differentiation of small (≤2 cm) well-differentiated hepatocellular carcinomas from regenerative and dysplastic nodules.

**Methods:**

Seventy-three cirrhotic patients, with 118 focal liver lesions (≤2 cm) were prospectively recruited. MRI examination was performed with a 3T magnet and the study protocol included T1 - and T2-weighted pre-contrast sequences and T1 -weighted gadoxetic-acid enhanced post-contrast sequences obtained during the arterial, venous, late dynamic and hepatobiliary phases. All lesions were pathologically confirmed. Two radiologists blinded to clinical and pathological information evaluated two imaging datasets; another radiologist analysed the signal intensity characteristics of each lesion. Sensitivity, specificity and diagnostic accuracy were considered for statistical analysis.

**Results:**

Good agreement was reported between the two readers (κ 0.70). Both readers reported a significantly improved sensitivity (57.7 and 66.2 vs 74.6 and 83.1) and diagnostic accuracy (0.717 and 0.778 vs 0.843 and 0.901) with the adjunction of the hepatobiliary phase 57.7 vs 74.6 and 66.2 vs 83.1 (*p* ≤ 0.04).

**Conclusions:**

Gadoxetic-acid MRI is a reliable tool for the characterization of HCC and lesions at high risk to further develop.

## Background

Hepatocellular carcinoma (HCC) occurs primarily in subjects with chronic liver disease or liver cirrhosis and is the cause of death in this population. The development of HCC may arise from de novo hepatocarcinogenesis or by means of a multistep progression from regenerative nodule, through dysplastic nodule to HCC [1, 2]. Especially for small nodules, it is sometimes very difficult to characterize a liver lesion due to the overlap of imaging features among different entities especially between dysplastic nodules and well-differentiated HCCs. Magnetic resonance imaging (MRI) has shown a poor diagnostic performance in this setting with a sensitivity ranging from 55 to 72 % [3, 4]. With the introduction of hepatobiliary system–specific contrast media in clinical practice, MRI can improve the detection and characterization of liver tumours with a sensitivity value ranging from 72 to 92 % [4–6]. Gadoxetic acid is a paramagnetic, gadolinium-based contrast medium that combines perfusion and hepatocyte-selective properties. In a single examination, gadoxetic acid enables the standard dynamic MRI study of the liver and the evaluation of the functional liver tissue, due to the uptake of approximately 50 % of the contrast agent by the hepatocytes [7]. The role of a liver specific contrast agent in the detection of HCC in cirrhotic liver has been well established in the literature, but definitive data on the role of MRI in the “grey zone” of the hepatocarcinogenesis (regenerative nodule, dysplastic nodule and well-differentiated HCC) are still partially lacking [8, 9]. The purpose of the present study is to distinguish well-differentiated HCCs from regenerative nodules and dysplastic nodules using gadoxetic acid MRI, mainly focused at the hepatobiliary phase.

## Methods

### Patient population

**Fig. 1:**
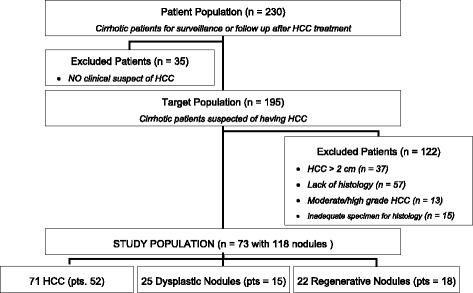
Flowchart of the enrolment of the study population based on recommended standards for reporting diagnostic accuracy and proof of tumour burden

This prospective study was approved by the Institutional Review Board of “Sapienza” University of Rome, Department Radoiological Sciences, Oncology and Anatomical Pathology and followed the principles of the 1964 Declaration of Helsinki and subsequent amendments. Informed consent was obtained from all individual participants included in the study. Between January 2014 and July 2015, 230 consecutive patients with chronic liver disease were evaluated prospectively within the Liver Unit of the Department of Gastroenterology for the examination of their pathology and the evaluation of suspected lesion at Ultrasound. Among these, 157 patients were excluded from data analysis for the following reasons: 1) absence of focal lesion at MR exam *n*° = 35; 2) focal lesion greater than 2 cm *n*° = 37; 3) lack of histologically proven lesion *n*° = 57; 4) inadequate specimen for pathological analysis at liver biopsy *n*° = 15; and 5) moderate/high grade of HCC n° = 13. The inclusion criteria were focal liver lesions ≤ 2 cm in diameter and their histological confirmation. The final study population was composed of seventy-three patients (47 males – 26 females; mean age 64 years; range 23–82 years), with 118 focal liver lesions (Fig. 1). Histological information had been obtained by liver transplantation, surgery and liver biopsy according to the best clinical care for the patient. Liver biopsy was mainly performed for the characterization of nodules with MRI patterns that were not suggestive of HCC (wash-in and wash-out), and in a few cases of HCC with typical aspects, a liver biopsy was performed before radiofrequency ablation. Patients who had undergone surgery or biopsy were followed by CT or MR examinations with a surveillance interval of 6 months [10]. Chronic hepatitis or cirrhosis were related to viral infection (hepatitis C [*n*° = 31], hepatitis B [*n*° = 13], both [*n*° = 2]), alcohol abuse (*n*° = 7), alcohol + HCV infection (*n*° = 11) or cryptogenic (*n*° = 9). Forty patients were Child-Pugh A classified, 22 were class B, and 11 were class C.

### MRI technique

MRI was performed using a 3 Tesla scanner (Discovery MR750; General Electric Systems, Milwaukee, Wisconsin, US) equipped with a high-performance gradient system (50 mT/m). Signal reception was achieved using a combined antero-posterior phased-array surface coil and a spine array coil. MRI sequences and parameters are detailed in Table 1.

**Table 1:** MR imaging sequences and parameters

MR Sequence	Fat suppression	TR/TE (OP/IP) (ms)	Flip angle (degrees)	Section thickness (mm)	Matrix size	Bandwidth (Hz/pixel)	Field of view (cm)	Time (s)
T2-weighted 2D SSFSE	w/ and w/out	3000/110	90	5	320 × 324	260	30–40	32
T1-weighted 2D dual GRE	Not used	4/1.2–2.4	12	5	320 × 324	260	30–40	32
SS-EPI-DWI	Used	5455/77	90	6	100 × 192	250	30–40	30
T1-weighted 3D GRE LAVA^a^	Used	4.2/1.3	12	5 (interpolated 2.5)	320 × 224	250	30–40	23

^a^Acquired before and after contrast medium administration during the arterial (≈25 s), venous (70 s.), late dynamic (180 s.) and hepatobiliary phases (20 min)

Timing for the initial post-contrast arterial phase acquisition was determined using an automated bolus detection technique (SmartPrep, General Electric) [11]. The full 0.025 mmol/kg body weight dose of gadoxetic-acid (Primovist Bayer Schering Pharma, Berlin, Germany: 0.1 mL/kg body weight) was administered at the flow rate of 2 mL/sec through an 18–22-gauge intravenous catheter by means of a power injector (Spectris; Medrad, Indianola, Pa), followed by a 20-mL saline flush at the same injection rate. Post contrast images were obtained ≈ 25, 70, and 180 s after contrast medium injection, during the hepatic arterial, hepatic venous, and late dynamic phases, respectively, as well as during the hepatobiliary phase (20 min after contrast medium administration). Before the administration of gadoxetic-acid, a respiratory-triggered single-shot echo-planar imaging DWI MR sequence was also acquired with b values of 0, 50, 400 and 800 s/mm2. A spectral attenuated inversion-recovery technique was used for fat suppression of DW images.

### Image analysis

One radiologist (with 3 years of experience in liver imaging), who was not involved in the data set analysis, evaluated the signal intensity of each lesion at all acquired sequences (precontrast, dynamic and hepatobiliary phases) as hyperintense, isointense, or hypointense relative to liver parenchyma. Two data sets of images were generated: a) pre-contrast sequences + dynamic phase sequences and b) pre-contrast sequences + dynamic phase sequences + hepatobiliary phase sequences.

All data sets of images were independently reviewed by two radiologist experts in abdominal MRI (C.C. 18 years, M.D.M. 10 years), in two reading sessions with a time interval of four weeks to avoid any recall bias, by using a commercially available workstation (Leonardo; Siemens Medical Systems) with standard interpretation tools (window width, pan, level). The readers were blinded to the results of histopathologic analysis. HCC was unequivocally diagnosed if it was hypervascular duting the hepatic arterial phase and fulfilled any one of the following five criteria: (a) hypointensity compared with the surrounding liver during portal venous or late dynamic phases (wash-out sign), (b) peripheral rim enhancement during the late dynamic phase (capsular appearence), (c) invasion line of adjacent vessels, and (d) hypointensity during the hepatobiliary phase. In addition, suggestive but non-conclusive criterion of HCC included (a) mild hyperintensity on T2-weighted MR images or (b) nodular early enhancement without washout.

### Pathologic analysis

All resected and explanted livers were analysed by the same experienced (30 years of experience) pathologist. They were sectioned in the axial plane with a slice thickness of 5–10 mm. The MRI images had been directly correlated with histological findings by an expert radiologist (10 years of experience in abdominal imaging) who was present when the specimens were prepared for evaluation. Percutaneous needle nodule biopsy was performed with an 18-gauge needle, under local anaesthesia and ultrasound guidance. Each biopsy specimen was approximately 1.5 cm in length. No complications, such as bleeding and/or seeding, were reported after liver biopsy. The diagnosis of hepatocellular nodules was performed according to criteria of the International Working Party on haematoxylin and eosin (H&E)-stained sections supplemented by CD34 immunostaining for nodule vascularization. Criteria for the differentiation of HCC from dysplastic nodule were tumour invasion into portal tracts (stromal invasion) and presence of multi-foci of neo-angiogenesis [12, 13].

### Statistical analysis

Inter-reader variability between the readers for lesion detection was assessed by using the weighted κ statistic. K values of 0.4 or less were considered positive but fair agreement, those of 0.41–0.60 moderate agreement, those of 0.61–0.80 a good agreement and greater than 0.80 indicated an excellent agreement [14]. The accuracy of each imaging method was determined using a jackknife alternative, free-response receiver operating characteristic (JAFROC_v3b_BETA), considering fixed readers and random cases [15]. The area under each curve (AUC) was used to indicate the overall diagnostic performance of each reader on each image set. Determinations of the sensitivity, specificity, and positive and negative predictive values (PPV and NPV, respectively) for lesion detection on each image set for each reader were calculated against reference standard findings. The 95 % confidence interval (CI) was also calculated for each evaluation. To consider the possible presence of multiple lesions within the same patient, the significance of differences in sensitivity and PPV among the different image sets was assessed using a generalized linear mixed model with P values calculated using an adjustment of the McNemar test [16]. Statistical analyses were conducted using dedicated software (SPSS version 13.0, SPSS, Chicago, Ill, US).

## Results

### Qualitative analysis

**Fig. 2:**
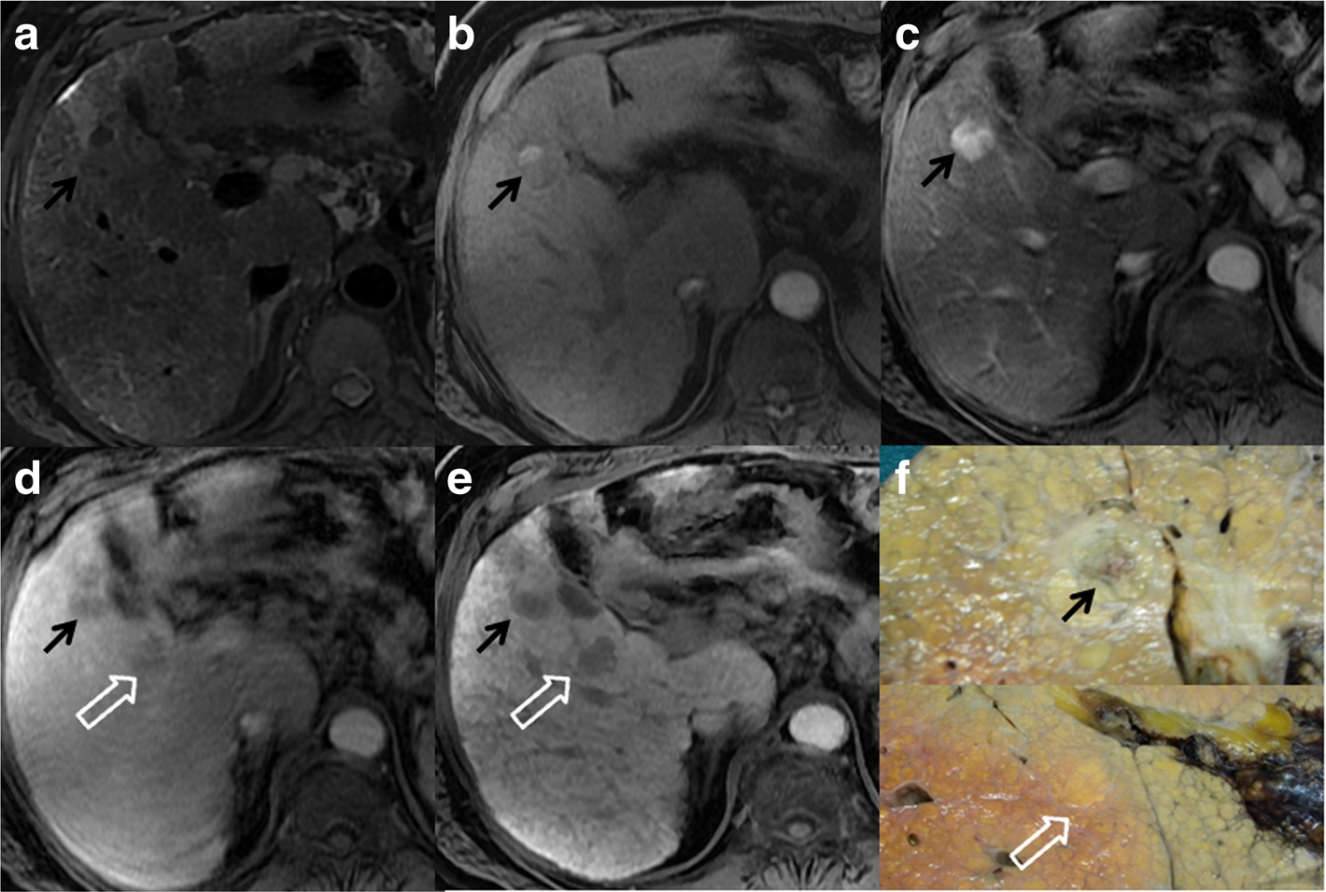
MR scans in a patient with chronic hepatitis, HCV related, and HCC and dysplastic nodule in liver segment V. **a**-**b** T2- and T1-weighted fast images show a heterogeneous nodule in liver segment V (*arrow*). The lesion shows the typical pattern of HCC with enhancement during the arterial phase **c**) and a wash-out sign on late dynamic phase **d**). On the late dynamic phase, a lesion near the “hilum-hepatis” is also detectable with loss of signal intensity to the surrounding liver parenchyma (open arrow). **e** On the fat-suppressed T1 -weighted 3D GRE image obtained during the hepatobiliary phase at 20 min after contrast injection, both lesions are hypointense to the surrounding liver parenchyma. **f** Histological analysis shows a hepatocellular carcinoma (*upper image*) and dysplastic nodule (*lower image*)

**Fig. 3:**
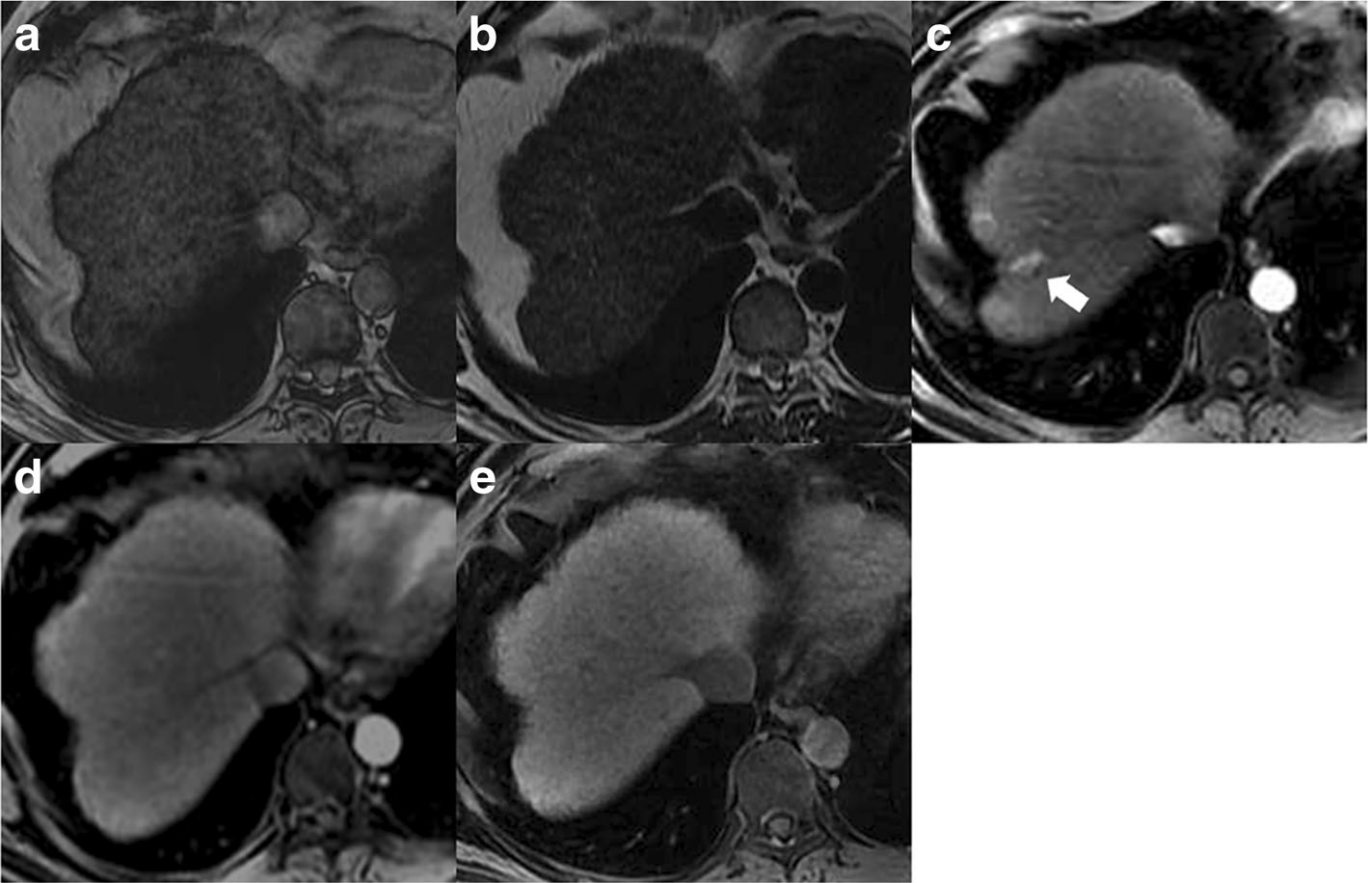
MR scans in a patient with chronic hepatitis, HCV related, and a regenerative nodule in liver segment VIII. **a**-**b** T1 - and T2-weighted images do not reveal any focal liver lesion. **c** T1-weighted gradient-echo shows a hypervascular lesion without wash-out during the late dynamic phase **d**). On the corresponding MR image obtained during the liver-specific hepatobiliary phase (**e**), the lesion is isointense to adjacent hepatic parenchyma. At pathologic examination of the explanted liver, this lesion corresponded to a multiacinar cirrhotic nodule

Among the 118 identified lesions (1 to 4 per patient), 71 in 52 patients were well-differentiated HCC (range 5–20 mm; median 15 mm) of which 32 were confirmed at liver transplantation, 15 at surgery and 24 at biopsy; 25 lesions in 15 patients were dysplastic nodules (range 5–20 mm, median 15 mm), of which 14 were confirmed at liver transplantation, 6 at surgery and 5 at biopsy. Additionally, 22 in 18 patients were regenerative nodules (range 6–20 mm; median 15 mm) of which 16 were confirmed at liver transplantation and 6 at biopsy. MRI appearance of regenerative, dysplastic nodules and well-differentiated HCC at different MRI sequences are summarized in Table 2. Well-differentiated HCC showed the typical imaging pattern (wash-in and wash-out) in 43/71 lesions (60.5 %), 16/71 (21.1 %) were hypervascular without wash-out and 12/71 (16.9 %) were hypointense during the arterial phase with loss of signal intensity during the late dynamic phase (Fig. 1). During the hepatobiliary phase, six typical HCCs showed uptake of the contrast medium (13.9 %); by contrast among hypervascular lesions without wash out, 7 nodules out of 16 (43.7 %) showed loss of signal intensity during the hepatobiliary phase, which means that there is an absence of functional hepatocytes and that it should be considered a sign of malignancy. All hypovascular HCCs reported a loss of signal intensity during the late dynamic phase, and four of these were isointense during the hepatobiliary phase. Additionally, 35 out of 71 (50.7 %) of the well-differentiated HCCs, mostly HCCs with the typical imaging pattern, were hyperintense on T2-weighted images. Data of Low Grade Dysplastic Nodules (LGDN) and High Grade Dysplastic Nodules (HGDN) were pooled because only 7 cases of LGDN out of 29 were identified. Dysplastic nodules, in most cases, appeared as a nodule without enhancement (hypo- or isointense) during the arterial phase and were relatively hypointense during the late dynamic phase 20/25 (80 %). Among these, 7 (40 %) were hypointense during the hepatobiliary phase (Fig. 2). Eight dysplastic nodules, confirmed at liver biopsy and showing a loss of signal intensity on both late dynamic and hepatobiliary phases, subsequently developed the typical imaging pattern of HCC (wash-in and wash-out) within 6–12 months. In four cases (16 %) confirmed at liver transplantation, dysplastic nodules demonstrated the typical pattern of HCC and represented the main cause of false positive calls. None of the dysplastic nodules were hyperintense on T2-weighted images and most of them (20/25, 80 %) were hypointense to the surrounding liver parenchyma: in effect at histological examination some iron particles were found within the nodules. Regenerative nodules tend to be isointense to liver parenchyma in all pre-contrast and post-contrast dynamic phases. Only 4 out of 22 nodules (18.8 %) were hypervascular during the arterial phase (Fig. 3) and 2 lesions were slightly hypointense during the delayed phase. One lesion out of two showed high signal intensity on hepatobiliary phase and none of the regenerative nodules reported low signal intensity during the hepatobiliary phase. The other regenerative nodules were localized because they were hyperintense on T1-weighted images 6/22 (27.3 %) and/or slightly hypointense on T2-weighted images 10/22 (45.5 %) and/or 10/22 (45.5 %) hyperintense on hepatobiliary phase. Diagnostic performance regarding the detection of HCC showed a good inter-reader agreement (κ .70) between the two observers. Both readers detected significantly more malignant lesions on MRI datasets that included pre-contrast, dynamic and hepatobiliary phases than on MRI datasets that included only pre-contrast and dynamic phases (57.7 and 66.2 vs 74.6 and 83.1 : *p* = 0.049 and *p* = 0.03) (Table 3). The overall accuracy for the detection of HCC was higher on dynamic + hepatobiliary phase MRI for both readers compared to dynamic MRI alone, and both radiologists reported a significant difference (0.717 and 0.778 vs 0.843 and 0.901: *p* = 0.03) (Table 3). The hepatobiliary phase was useful for a definitive diagnosis of malignancy in 7 of 16 cases (43.7 %): these nodules showed enhancement during the arterial phase and no wash-out sign during the venous and late dynamic phase.

**Table 2:** Signal intensities of different lesions at each MR sequence

	T1-w	T2-w	T1-Art	T1-Ven	T1-LD	T1-Hepatobiliary
Well diff. HCC -	57,7 hyper (27/45)	53.3 hyper (24/45)	82.2 hyper (37/45)	64.4 iso (29/45)	66.6 hypo (30/45)	84.2 hypo (38/45)
D.N. -	55,1 hyper (16 /29)	58.6 hypo (17/29)	58.6 iso/hypo (25/29)	68 hypo (17/29)	89.6 hypo (26 /29)	58.6 iso/hyper (17/29)
R.N. -	51 iso / hyper (17/33)	75.7 iso (25/33)	57.7 iso (19/33)	87.8 iso (29/33)	84.8 iso (28/33)	100 iso / hyper (33/33)

**Table 3:** Diagnostic performance for HCC detection

		Sensitivity	Specificity	Positive predictive value	Negative predictive value	Accuracy
Observer 1	Dynamic phases	57.7 %(41/71) [.45–.69]	91.5 %(43/47) [.80–.97]	91.1 %(41/45) [.78–.97]	58.9 %(43/73) [.49–.71]	0.717 [.60–.83]
	Dynamic + hept phases	*74.6 %(53/71) [.61–.83]	91.5 %(43/47) [.80–.97]	92.9 %(53/57) [.79–.97]	70.5 %(43/61) [.53–.75]	^§^0.843 [.73–.95]
Observer 2	Dynamic phases	66.2 %(47/71) [.54–.77]	91.5 %(43/47) [.80–.97]	92.2 %(47/51) [.85–.98]	62.7 %(42/67) (54/85)	0.778 [.64–.88]
	Dynamic + hept phases	*83.1 %(59/71) [.72–.91]	95.7 %(45/47) [.85–.99]	96.7 %(59/61) [.87–.98]	78.9 %(45/57) [.52–.73]	^§^0.901 [.78–.99]

Numbers in brackets are the 95 % CIs

*Significantly higher sensitivity for both readers *p* = 0.04 and *p* = 0.03

^§^Significantly higher sensitivity for both readers *p* = 0.03

Both the PPV and NPV for HCC identification were higher on dynamic + hepatobiliary phases MRI compared to dynamic phases MRI alone (Table 3).

## Discussion

Our experience confirmed that Gadoxetic acid MRI is a reliable tool for the identification of HCC or lesions at high risk of developing into HCC. Loss of signal intensity during the hepatobiliary phase helps to improve the detection of small (≤20 mm) hypervascular well-differentiated HCCs without washout during the dynamic phases. With the development of fast sequences and the acquisition of images at different vascular phases, MRI has demonstrated a trend to a better sensitivity and accuracy over CT, and with the introduction in clinical practice of liver specific contrast agent, this difference has become more significant [17, 18]. At present, indeed, MRI is considered the best imaging approach in the evaluation of nodules in cirrhotic patients. However, the detection and characterization of small HCC in cirrhotic patients is still challenging because it is difficult to distinguish HCC from other entities that could develop in cirrhotic liver. Considering imaging patterns of the three groups of lesions that were mentioned in the study (well-differentiated HCC, dysplastic nodule and regenerative nodule) at dynamic phases, HCCs tend to have a typical imaging pattern in 60.5 % of cases. In 14 out of 71 cases (19 %), HCC appears as isointense lesions with wash-out signs. This imaging pattern suggests that tumour neo-angiogenesis starts after the disruption of peri-portal space [19, 20]. The main feature of dysplastic nodules was hypovascular lesion (iso/hypointense) with a loss of signal intensity during the late dynamic phase. This is probably because in the dysplastic nodules there are only a few foci of neo-angiogenesis and the blood is drained to the sinusoid by the surrounding liver parenchyma, but it could also be explained by the early uptake of the gadoxetic acid from the liver parenchyma near the lesion. Ten dysplastic nodules showed a loss of signal intensity on both late dynamic and hepatobiliary phases. These image findings overlapped in our study population with some hypovascular HCCs. Eight of those dysplastic nodules changed their imaging patterns to that of HCC at follow-up imaging. Loss of signal intensity on both the late dynamic and hepatobiliary phase should then be considered a high feature of malignancy and could predict malignant transformation, and a more intensive management would be required (strict follow-up or biopsy) [21–23]. As suggested by previously published papers, in our experience, gadoxetic-acid MRI significantly increases sensitivity and diagnostic accuracy in the detection of small hepatocellular carcinoma. [24–27]. In our experience, a high signal intensity on T2 should be certainly considered a sign of malignant transformation because it was encountered only in HCCs, although with a poor detection rate (53.3 %) [28]. Our study certainly has some limits. Firstly, in our study we considered low grade and high grade dysplastic nodules in the same group due to the small number of low grade dysplastic nodules. We are aware that there is a significant difference between these two entities in developing HCC. Secondly, some histological confirmations were obtained with liver biopsy, which in small nodules may lead to a small amount of sampling tissue and difficult nodule characterization. Finally, although DWI MRI sequence is a part of the MRI protocol at our institution, data were not reported in this paper because the preliminary results showed no significant differences of adding DW images for the detection of HCC. The role of DW images in cirrhotic liver is still a matter of debate. Some authors emphasize that it is useful for the detection and characterization of nodules in cirrhotic liver [29], while others affirm that it only slightly increases MRI sensitivity [30, 31].

## Conclusion

In our experience, loss of signal intensity at gadoxetic acid MRI hepatobiliary phase is a reliable tool in the identification of HCC and lesions at high risk to develop into HCC. Cirrhosis-associated hepatocellular nodules with non specific findings at MR imaging, that show loss of signal intensity during the hepatobiliary phase should undergone more intensive management.

## Abbreviation

DN: Dysplastic nodule
DWI: Diffusion weighted imaging
HCC: Hepatocellular carcinoma
HGDN: High grade dysplastic nodules
LGDN: Low grade dysplastic nodules
MRI: Magnetic resonance imaging
RN: Regenerative nodule
